# Microbial flora, probiotics, *Bacillus subtilis* and the search for a long and healthy human longevity

**DOI:** 10.15698/mic2017.04.569

**Published:** 2017-03-16

**Authors:** Facundo Rodriguez Ayala, Carlos Bauman, Sebastián Cogliati, Cecilia Leñini, Marco Bartolini, Roberto Grau

**Affiliations:** 1 Departamento de Microbiología, Facultad de Ciencias Bioquímicas y Farmacéuticas, Universidad Nacional de Rosario. CONICET - Rosario. Argentina.

**Keywords:** Bacillus subtilis, probiotics, biofilms, lifespan, healthy longevity, dietary restriction, insulin signaling

## Abstract

Probiotics are live microorganisms that have beneficial effects on host health, including extended lifespan, when they are administered or present in adequate quantities. However, the mechanisms by which probiotics stimulate host longevity remain unclear and very poorly understood. In a recent study (*Nat. Commun.* 8, 14332 (2017) doi: 10.1038/ncomms14332), we used the spore-forming probiotic bacterium *Bacillus subtilis *and the model organism *Caenorhabditis elegans* to study the mechanism by which a probiotic bacterium affects host longevity. We found that biofilm-proficient *B. subtilis* colonized the *C. elegans* gut and extended the worm lifespan significantly longer than did biofilm-deficient isogenic strains. In addition to biofilm proficiency, the quorum-sensing pentapeptide CSF and nitric oxide (NO) represent the entire *B. subtilis* repertoire responsible for the extended longevity of *C. elegans*. *B. subtilis* grown under biofilm-supporting conditions synthesized higher levels of NO and CSF than under planktonic growth conditions, emphasizing the key role of the biofilm in slowing host aging. Significantly, the prolongevity effect of *B. subtilis* was primarily due to a downregulation of the insulin-like signaling system that precisely is a key partaker in the healthy longevity of human centenarians. These findings open the possibility to test if the regular consumption of *B. subtilis* incorporated in foods and beverages could significantly extend human life expectancy and contribute to stop the development of age-related diseases.

Why do people die? Leaving aside deaths produced in violent events such as accidents, armed robberies, terrorist attacks and armed conflicts, people die because of two “natural” causes: disease and/or aging. In the first case we state that a person died as a consequence of a particular disease process, in the second case we state that a person died because he/she aged. During the past century, scientists fought the first cause of natural death: human diseases. In 1918 the influenza pandemic or Spanish flu (1918-1919) infected 500 million people around the world, killed 50 to 100 million people and produced a drop of twelve years in life expectancy. Today is almost impossible to think of that amount of people killed because the outbreak of a pandemic with the characteristics of the Spanish flu. This is because humankind developed medicines, antibiotics, vaccines and protocols to prevent and control disease dissemination. For instance, the recent West African Ebola outbreak (2013 - 2016) was contained in the countries where it originated and caused around 11,000 deaths, a significant number of deaths but far below the ones produced by the 1918 flu pandemic. If the 2013 Ebola outbreak would have appeared one century ago it would had produced deaths by hundreds of millions. Therefore, we could agree on the point that scientists have given a hard blow to the first cause of natural death: disease, mainly infectious diseases.

A dramatic increase of 5 years in life expectancy happened between 2000 and 2015, although major inequalities still occur all around the world. Global life expectancy for children born in 2015 is reaching 72 years and the children who will reach 120 - 125 years of age are walking between us but nobody knows who they are. The top five countries possessing the highest life expectancy (an also healthy life expectancy) are Japan, Switzerland, Singapore, Australia and Spain (average life expectancy of 83.16 years). During the present century, scientists are fighting the second cause of natural death (aging) hoping to extend human healthy longevity further than centuries.

How do we fight aging? Aging is a multifactorial and poorly understood process characterized by progressive impairment of the host response to stresses and general cellular deterioration of key metabolic pathways. Intensive work in different animal models (i.e., hydras, yeasts, worms, flies, mice and monkeys) has identified a number of factors that promote longevity. Restricting food intake (DR), decreasing insulin/IGF-1 signaling (IIL), slowing mitochondrial respiration, reducing germline function, or lowering temperature can all extend lifespan. In this respect, *Caenorhabditis elegans* is probably the most suitable model organism for research on aging because this simple and translucent nematode has regulatory and metabolic pathways related to aging conserved throughout evolution.

Recent reports focused on the existence of a positive relationship between human health and the microbial flora that colonize the human gut. Among the gut bacteria, which behave as a functional human organ, probiotics represent the Holy Grail because they are associated with a broad spectrum of positive effects on host health, including positive effects on host longevity. However, the mechanisms by which probiotics affect host longevity remain unclear. In our recent publication (*Nat. Commun*. 8, 14332 (2017) doi: 10.1038/ncomms14332) we used the probiotic bacterium *Bacillus subtilis *and the model organism *C. elegans* to understand the mechanism by which probiotic biofilm affects host longevity.

How do probiotic bacteria interact with the host? Bacteria do not live isolated as individual and self-sufficient creatures in nature. On the contrary, bacteria live in natural settings as multicellular and cooperative (social) communities called biofilms or cities of microbes. These biofilms are three-dimensional structured communities of adherent microorganisms encased in a self-produced extracellular matrix, containing networks of channels for nutrient supply and long-distance cell-to-cell communication (quorum sensing, QS) used for division of labor between members of the community. Several reports have shown the importance of biofilms for the success of pathogens in the infection process in different animal models, a finding that also applies to the biofilms of human pathogens. However, very little is known about the role of the biofilm produced by beneficial bacteria during the interaction with the host.

Our results indicated that biofilm-proficient *B. subtilis* colonized the *C. elegans* gut and extended the worm lifespan significantly longer that did biofilm-deficient isogenic strains. Bacteria living in a biofilm are physiologically very distinct from their planktonic counterparts, and they function as a cooperative consortium more similar to that of multicellular organisms than a unicellular organism. In addition to biofilm proficiency, the quorum-sensing pentapeptide PhrA or CSF (needed for efficient cell to cell communication) and nitric oxide (NO) represent the entire *B. subtilis* repertoire responsible for the extended longevity and health of *C. elegans*. *B. subtilis* grown under biofilm-supporting conditions synthesized higher levels of NO and CSF than under planktonic growth conditions, emphasizing the key role of the biofilm in slowing host aging. These results directly implicate the probiotic biofilm as the primary cause of the increased lifespan and healthy longevity of *C. elegans* when fed the probiotic bacterium *B. subtilis*. This dual microbial-worm interaction would allow the bacterium to colonize and establish a multicellular biofilm in the friendly environment of the worm gut mucosa, a similar scenario to the formation of stable biofilms during the beneficial bacteria-plant interaction of some Bacilli (i.e., *B. subtilis* and *B*. *amyloliquefaciens*) with PGPR (Plant Growth Promoting Rhizobacteria) activity on plant roots (rhizospheric biofilm).

How is the prolongevity effect of *B. subtilis* transduced through the sensory pathways that regulate aging? Lifespan is subject to regulation by conserved signaling pathways and transcription factors that sense stress, environmental cues and nutrient availability. DR and the insulin-like signaling (ILS) pathway are central for the regulation of longevity in different animal models, including *C. elegans* and humans. These longevity regulatory pathways converge on the positive and negative regulation of the transcription factors DAF-16 (FOXO in humans) and HSF-1, respectively. Significantly, the prolongevity effect of *B. subtilis* was primarily under DAF-2 (IGF-1 in humans)/DAF-16/HSF-1 control, a finding that links extended lifespan to downregulation of the insulin-like signaling (ILS) pathway. Interestingly, healthy human centenarians likely have IGF-1 receptor genetic variants associated with a slightly reduced functionality of the insulin signaling, an intriguing observation that positively correlates with our results.

This year marks the centennial of the death of Elie Metchnikoff, the father of innate immunity. In 1907, he was the first to propose the concept of probiotic bacteria, hypothesizing that probiotic lactic acid bacteria (LAB), mainly found in yogurt, were important to promote human health and longevity. He noticed the unusually high longevity of some residents of Eastern Europe in comparison with people living in Western Europe or U.S. Many of the centenarian people whom he analyzed were poor, with very simple lifestyles, but who consumed large amounts of yogurt containing *Lactobacillus bulgaricus*. Because of these and other observations, Metchnikoff proposed that human aging was the result of intestinal microbe dysbiosis (unbalanced gut flora) and that consumption of probiotic LAB (i.e., consuming yogurt) could delay senility (i.e., enhance healthy longevity) because of the reestablishment of a healthy gut flora, an interesting hypothesis that at that time did not receive further attention. The advantage of probiotic Bacilli over LAB primarily relies on two beneficial properties for human consumption: refrigeration is not required for maintaining Bacilli viability (because they produce tough spores), and these bacteria can be added to a wide range of foods and beverages in addition to dairy products. DR is the only non-genetic intervention that extends lifespan in mammals, but extending its benefits to human longevity is unlikely because DR is hard to follow and accomplish. Our work showed that consumption of *B. subtilis* enhances host longevity without genetic intervention and raised the possibility to feed people with probiotic *B. subtilis* incorporated in different foods and beverages regardless if they are cheap or expensive and without affecting cultural traditions.

**Figure 1:**
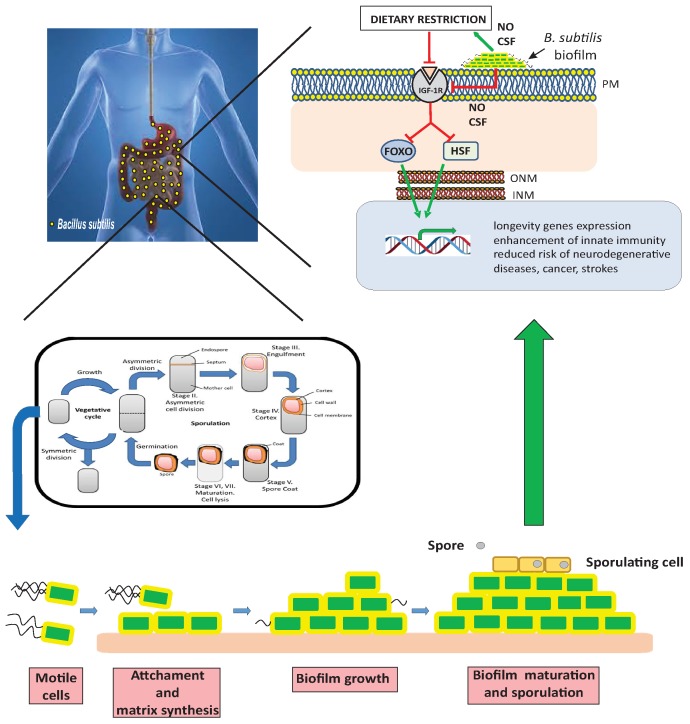
FIGURE 1: A workable model of how probiotic *B. subtilis* improves host health and longevity. Once spores of the probiotic bacterium *B. subtilis* are incorporated in the diet and consumed, they survive the transit through the stomach and reach the human intestine **(Left cartoon)**. These gut spores germinate and the active form of the probiotic (vegetative cells of *B. subtilis*) emerges, multiples and forms a beneficial biofilm in the host intestine **(Bottom cartoon)**. Biofilm *B. subtilis* cells produce a continued and coordinated provision of beneficial and anti-aging NO and CSF molecules to the host tissues (bottom and right cartoons). At genetic level, longevity is regulated by the activity of the gene-transcription factors FOXO and HSF **(Right cartoon)**. The binding of insulin-like molecules activates insulin receptor which in turn activates a series of protein kinase enzymes that phosphorylate FOXO, keeping it inactive in the cytoplasm. Additionally, active insulin receptor is responsible for the formation of an inhibitory protein complex that sequesters HSF in the cytoplasm. Beneficial signals (NO, CSF and others) derived from the biofilm established by *B. subtilis* produce a direct or indirect (through DR activation) downregulation of insulin receptor **(Right cartoon)**. Upon downregulation of the insulin receptor, FOXO and HSF become active in the nucleus. There, both prolongevity transcription factors orchestrate the activation of host genes responsible for (i) resistance to age-related diseases and (ii) a prolonged and healthy longevity. Symbols: (red) repression, (green) activation.

Could probiotic *B. subtilis* extend human life expectancy? Ranking at the top of human welfare, Japan exhibits the highest world longevity (84 years and 81 years, for female and male, respectively), and home to more than 65,000 centennial persons. Together with a longer longevity, a good quality of life and strong health are desired in elderly people. Japan also exhibits the highest healthy life expectancy (78 years for both sexes at the time of birth). What is the secret of the healthy Japanese longevity? Aging depends on genetic and environmental factors, including dietary habits. In the regular diet of the Japanese population exists the millenarian food called natto (“vegetable cheese”), a natural food that consists of soybean fermented by cells of *B. subtilis*. Because *B. subtilis* is the active ingredient of this popular and ancient food, it is tempting to pay attention to this probiotic bacterium, which might naturally contribute to the long and healthy longevity of Japanese people. Taking into consideration that probiotic Bacilli can be incorporated in a daily and safe dose (i.e., 1.0-2.0 x 10^9^ spores/day) in many types of human foods and beverages; the centenarian Metchnikoff hypothesis; and our results, it might be worth investigating whether the regular consumption of probiotic* B. subtilis* in human food might decrease the rate of aging and detect and stamp out disease because of downregulation of insulin/IGF-1 signaling and enhancement of innate immunity, respectively, at the earliest possible moment. Future studies will address the validity of this provocative hypothesis, elucidate the detailed biochemical mechanism responsible for *Bacillus*-induced ILS/DR-dependent long longevity and its contribution to the current war against the second cause of natural death: aging (Figure 1).

